# Aspects of structural health and condition monitoring of offshore wind turbines

**DOI:** 10.1098/rsta.2014.0075

**Published:** 2015-02-28

**Authors:** I. Antoniadou, N. Dervilis, E. Papatheou, A. E. Maguire, K. Worden

**Affiliations:** 1 Dynamics Research Group, Department of Mechanical Engineering, The University of Sheffield, Mappin St., Sheffield S1 3JD, UK; 2 Vattenfall Research and Development, New Renewables, The Tun Building, Holyrood Road, Edinburgh EH8 8AE, UK

**Keywords:** structural health monitoring, condition monitoring, offshore wind turbines, data analysis

## Abstract

Wind power has expanded significantly over the past years, although reliability of wind turbine systems, especially of offshore wind turbines, has been many times unsatisfactory in the past. Wind turbine failures are equivalent to crucial financial losses. Therefore, creating and applying strategies that improve the reliability of their components is important for a successful implementation of such systems. Structural health monitoring (SHM) addresses these problems through the monitoring of parameters indicative of the state of the structure examined. Condition monitoring (CM), on the other hand, can be seen as a specialized area of the SHM community that aims at damage detection of, particularly, rotating machinery. The paper is divided into two parts: in the first part, advanced signal processing and machine learning methods are discussed for SHM and CM on wind turbine gearbox and blade damage detection examples. In the second part, an initial exploration of supervisor control and data acquisition systems data of an offshore wind farm is presented, and data-driven approaches are proposed for detecting abnormal behaviour of wind turbines. It is shown that the advanced signal processing methods discussed are effective and that it is important to adopt these SHM strategies in the wind energy sector.

## 1. Introduction

Offshore wind farms are remotely located and operate under challenging conditions, much worse than the onshore wind farms. Early failure of their components has been frequently observed in the past. This fact could inhibit their establishment as an attractive alternative option for power generation. It is for this reason that the development of a reliable structural health monitoring (SHM) and condition monitoring (CM) strategy is particularly necessary and why current research is focusing on this aim.

In order to perform a health monitoring strategy, one of the initial steps is to acquire the appropriate data for the purpose of damage detection. For an online approach, which is probably more appropriate when dealing with remote structures, vibration-based monitoring has been proven to be a suitable choice. It can be effective with possibly small computational costs when using suitable methods. There have been several successful SHM and CM vibration-based analysis applications in the past. Generally, one could adopt data-based approaches or physics-based approaches, both having certain advantages and drawbacks [1]. Concerning the data-based methods that will be discussed here, the major issue seems to be that for some SHM and CM scenarios only advanced signal processing techniques would be able to deliver the desirable results. This is because of the fact that in the real world most of the times the features obtained from the monitored signals are, in general, also sensitive to changes caused by environmental and operational conditions [2]. In the case of offshore wind turbines, turbulence—a term that describes the stochastic scale-invariant changes in the flow properties of the air—and, most importantly, the effects of the operational wind turbine control system could influence the vibration signals obtained. So any technique adopted, in order for it to be effective, should be able to take into account these challenging characteristics of the signals and separate them from any available damage sensitive feature. Such techniques are discussed in §2 of this paper where some application examples are also demonstrated. These applications are focused mainly on wind turbine gearbox and blade damage detection, because it has been observed that these components have the most frequent failures and can cause the highest downtime in a wind turbine. Still, there is no limitation in the application of the methods to different components.

The paper also deals with the manipulation of a different kind of data coming from wind turbines: data obtained by the supervisor control and data acquisition system (SCADA) systems installed in many wind farms nowadays. They contain measurements of various variables, such as wind speed, bearing and oil temperatures, voltage and the power produced, among others. The recordings of these systems are constant and available for every wind turbine in a farm, so they could be potentially used to monitor wind farms. Therefore, exploitation of these measurements that could lead to an effective online SHM plan seems to be an attractive opportunity. In addition, because of the availability of such data for every individual wind turbine, one could explore novel approaches in the SHM field by treating the whole farm as a population. Exploring the potential of a population-based approach to damage detection in this case could refer to adopting strategies that can determine the condition of a wind turbine according to the measurements obtained from other wind turbines in the farm. In the corresponding section of the paper, the authors present some results that seem to be promising for further exploration of whether good predictions for individual wind turbines can be performed according to the wind turbine power curves constructed for each wind turbine, using some advanced machine learning.

Briefly, the aim of this paper is to highlight some of the most appropriate latest technologies to be applied for the SHM and CM of wind turbine systems.

## 2. Condition monitoring and structural health monitoring approaches for wind turbines

In the Introduction, a major problem when trying to perform online SHM and CM for data-based methods was mentioned: the influence of the varying conditions under which the wind turbines operate. This means simply that one should account for the non-stationarity that is not related to damage of the signals analysed, but this is definitely not a simple task. Vibration data coming from wind turbine blades and gearboxes while in operation should be influenced by the varying load and environmental conditions. Despite the fact that there has been significant research of damage detection methods for such components, most of the studies are mainly performed in the laboratory environment [3]. Basic signal processing techniques in this case might prove sufficient to solve the damage detection problem, although, in general, this would not be true in real-world damage detection scenarios.

Some of the most simple *time-domain methods* that have been used for condition monitoring are time-invariant analysis methods, based on the estimation of statistical parameters. This is a group of methods that uses univariate features. The principle of these methods is often to simply use the overall vibration level to describe the general condition of the machine. Estimates used in this case are peak amplitude, peak-to-peak value, root mean square value of the signal, crest factor and statistical moments such as variance, skewness and kurtosis [4,5]. More advanced techniques use multivariate statistics for damage detection or damage prognosis, and therefore do not belong to the category of time-domain methods [6–8].

Another technique, applied specifically to gear vibration signals, is based on the envelope estimation of the signals analysed. In these studies, amplitude and phase demodulation techniques are used to detect fatigue cracks in gears from the estimated envelope and instantaneous phase [9,10]. Most commonly in this case, the Hilbert transform (HT) is used in order to extract the instantaneous amplitude and phase of the vibration signal from the analytic signal (phase unwrapping). In addition, for the diagnostics of rolling element bearings, it has been shown that the analysis of the envelope signals is recommended, because the analysis of the raw signals does not always give enough information [11].

The ideas of time-series analysis have also been applied in condition monitoring. Useful features, in this case, can be generated if one identifies a good time-series model for the CM system examined when it is undamaged. High variance of the error signal (residual) between the model outputs and measured outputs shows that the system is damaged, because the model failed to make good predictions [12]. This approach is sometimes known as the time domain averaging method [13].

Other classic signal processing approaches can be grouped into what is known as *transformed domain methods*. These consist of the application of the Fourier analysis or cepstrum analysis in order to estimate the spectrum or cepstrum of the signals. The spectrum/cepstrum of the damaged gearbox is compared with its spectrum/cepstrum under the normal (undamaged) condition, and appropriate filtering can also be applied in order to isolate frequency bands believed to be associated with specific kinds of faults. Envelope analysis can also be performed in the transformed domain, in order to enhance the features examined. An example of the application of these methods is given in references [14,15]. Alternatively, the use of parametric models, such as the autoregressive (AR) or the autoregressive with exogenous inputs (ARX) models that can be used to estimate frequency domain features using the harmonic probing algorithm [16] can also be applied [17].

All the above methods, which most of the time use Fourier analysis, are considered to be classic signal processing techniques. But, because the Fourier transform (FT) does not explicitly reflect a signal's time-varying nature because it requires integration all over time and usually the vibration signals being analysed change over time, the FT and as a consequence the classic frequency domain approach for signal processing of non-stationary signals is generally inadequate. Because of this issue, most of the previously described methods might probably fail to detect damage if used on online measurements coming from a wind turbine. An alternative category of methods that could be used in this case are the *time-frequency* or *time-scale analysis methods*. These methods are more appropriate for damage detection in real-life scenarios because they can demonstrate the time-varying nature of the signals analysed. The simplest time-frequency method is the short-time Fourier transform (STFT). Comparing the signal being analysed with elementary functions that are localized in the time and frequency domains is the basic idea behind the method. The wavelet transform (WT) is another well-known *time-scale method*, having the same concept as the STFT. The major difference between the two methods is in the different basis/elementary functions chosen during the process that lead to different signal representations. Because of that, wavelets give better localization properties at high frequencies and are useful for detecting local events in the signals. Wavelet analysis is probably the most popular technique; a review of condition monitoring applications is found in reference [18]. In terms of an online implementation, the application of time-scale methods is less costly than a fast Fourier transform (FFT). In fact, the discrete wavelet transform can be carried out in O(*N*) operations compared with O(*N* log*N*) for the FFT. Some examples of WT applications in condition monitoring are given in [19–23].

Other common time-frequency methods are Cohen class time-frequency distributions such as the Wigner–Ville (WVD) and the Choi–Williams (CWD) distributions. The energy distribution in the joint time-frequency domain, such as the WVD, is very complicated as the underlying transforms are nonlinear. Condition monitoring applications of the WVD can be found in references [24–27].

Relatively recently, the empirical mode decomposition (EMD) method has also been proposed [28] as a filter bank method that can be applied for time-frequency analysis in combination with an AM–FM demodulation method such as the HT. This technique decomposes the signal into a number of meaningful signal components, representing simple oscillatory modes matched to the specific data. This is one of the basic advantages of the EMD when compared with other time-frequency methods. It has been applied since then in various condition monitoring applications [29–32].

At this point, an example of a time-frequency analysis of real wind turbine gearbox datasets is presented briefly. Owing to the limited space, only the major steps will be explained, but more details concerning this application can be found in [33]. The experimental gearbox vibration data analysed in this study come from an NEG Micon NM 1000/60 wind turbine in Germany. The measurements were taken by members of the company EC Grupa, a Polish engineering company that maintains the wind turbine system from which the gearbox vibration datasets were obtained.

The gearbox examined in this case consists of three gear stages: one planetary gear stage and two spur gear stages. The measurements come from a single accelerometer and were captured at a sampling frequency of 25 000 Hz. Acceleration signals from this gearbox were obtained at three different dates: 31 October 2009, 11 February 2010 and 4 April 2010. The gearbox was already known to have damage described to be at an initial state on 31 October 2009, but this dataset can still be used as a reference and could be useful in order to explore whether any damage-sensitive features change according to the development of the damage. The damage was ascribed to a damaged tooth at the second parallel gear stage.

**Figure 1.**
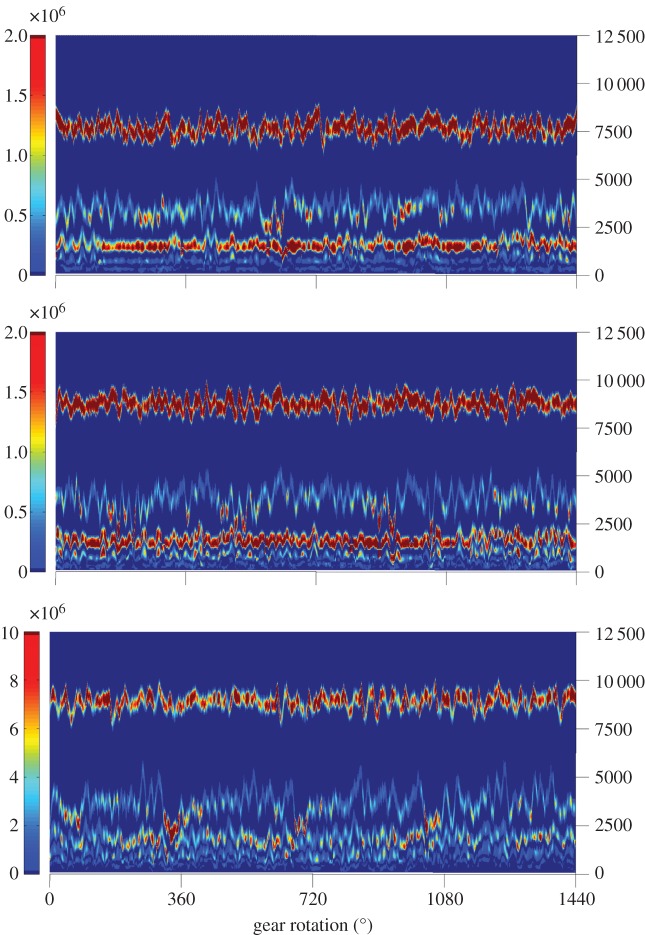
The Hilbert spectra of gearbox datasets at three different dates: (*a*) shows the spectra of 31 October 2009, (*b*) shows the spectra of 11 February 2010 and (*c*) of 4 April 2010. The horizontal axis shows the sample point, and the vertical axis shows the instantaneous frequency.

**Figure 2.**
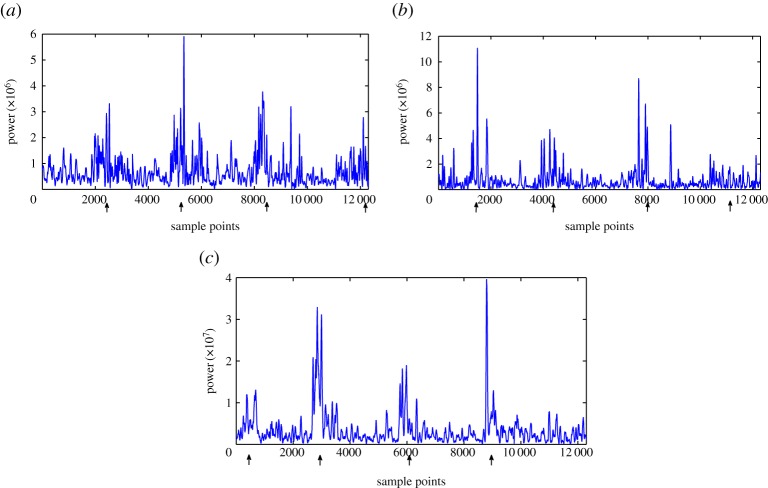
Power of the second IMF of the gearbox datasets. (*a*) Dataset 31 October 2009. (*b*) Dataset 11 February 2010. (*c*) Dataset 4 April 2010.

The EMD method was applied to the datasets, resulting in the creation of several intrinsic mode functions (IMFs; oscillatory functions/signal components) for each case. Then, using the HT method, the instantaneous amplitude and instantaneous frequency for each IMF was obtained, resulting in the time-frequency representation of the three different datasets shown in figure 1. Knowing that each IMF represents specific frequencies of the signal, and taking into account the meshing frequencies and their harmonics of the gears, one can expect that damage sensitive features should appear around the meshing frequencies and/or their harmonics of the damaged gear stage. The results agree with this concept, because the second IMF of the Hilbert spectra shown in figure 1 which is related to the previously discussed frequency bands, contains the desirable features. This is shown better in figure 2: the power diagrams of the second IMF are drawn individually for each case. One can observe that damage can be seen as an increase in the power of the specific IMF each time that the damaged gear tooth engages during the gear rotation.

An alternative approach that could be adopted in order to deal with the issue of non-stationarity when performing damage detection has been proposed in [2,34] for performing SHM of bridges. *Co-integration analysis*, a technique taken from econometrics, has been successfully applied for the purpose of removing environmental and operational trends in the vibration signals. The mathematics of the process are rather complex, but the main concepts of the method are summarized in the following: co-integration is a property of some non-stationary multivariate time series; an *n*-dimensional time series is co-integrated if some linear combination of the component variables is stationary. The combination is called a co-integrating relation, and the coefficients form a co-integrating vector. The analysis approach is largely based on the Johansen procedure, which is able to find the co-integrating vector that will result in the most stationary combination of the variables. Although the technique has not been yet applied on actual wind turbine data, an initial study describing how the method can be used for condition monitoring of wind turbine gearboxes was demonstrated in [33]. In this research, the results were promising for a successful application of the method in the wind turbine CM and SHM field.

What has been explained so far mostly summarizes feature extraction approaches, and mostly gearbox applications have been discussed. In the following, the focus is on blades applications with a pattern recognition perspective. In addition, the important matter of the sensor technologies used for blades is also discussed.

Wind turbine blades are susceptible to multiple modes of failure. Moreover, owing to the varying operating conditions observed in wind turbines and discussed earlier, a damage detection scheme is rather challenging. Another major issue is the appropriate choice of sensing technologies of the blades that could be applied in order to achieve a reliable online SHM system.

Some of the related research studies use either passive or active sensing technologies in the context of wind turbine blade SHM [35]. The difference between the two sensing approaches is that in passive sensing techniques there is no external/artificial excitation as is the case in active sensing techniques. In [36,37], an overview of sensor diagnostics for active sensing SHM systems by using piezoelectric transducers is given. In addition, an investigation of the optimal demarcation date that is essential for the proper normalization of active sensing data during their collection is presented.

Despite the fact that many SHM techniques and sensing technologies have been discussed in the literature for damage detection in blades, there has not been much progress on robust, online applications of these techniques in the SHM of in-service wind turbine systems. Some of the methods that have been applied in references [35,38–44] include vibration monitoring-based methods (accelerometers, piezo or microelectromechanical systems (MEMSs), strain (strain gauge or fibre optic cables), ultrasonic waves which are popular with composite structures (piezoelectric transducer), smart paint (piezoelectric or fluorescent particles), acoustic emissions (usually barrel sensors), impedance techniques (piezoelectric transducer), laser vibrometry (scanning laser Doppler), impedance tomography (carbon nanotube), thermography (infrared cameras), laser ultrasound (laser devices), nanosensors (electronic nanoparticles) and buckling health monitoring (piezoelectric transducer). In addition, wireless systems seem to be a good solution in the case that structures are remotely placed such as wind turbines. The main disadvantage of wireless sensors for SHM is the high demands for power supply of the sensors. This disadvantage is the reason for an increased interest in data telemetry with energy harvesting [45,46].

**Figure 3.**
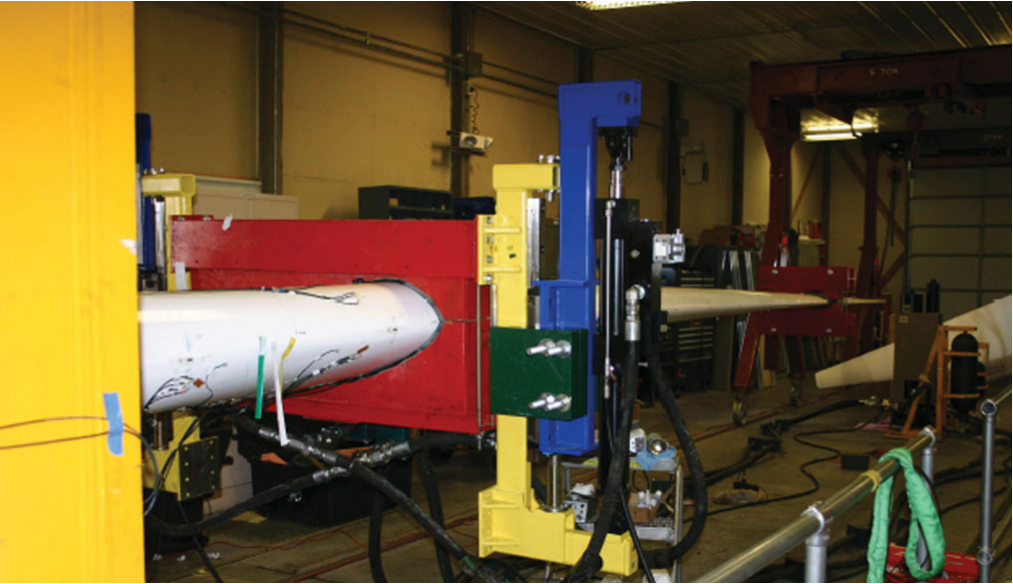
Wind turbine blade experiment under continuous fatigue loading [3].

Recently, a series of research studies around an experimental test of a 9 m wind turbine rotor blade (figure 3) that was dynamically loaded in a fatigue test until reaching catastrophic failure was conducted. The blade experiment was performed in the National Renewable Energy Laboratory and National Wind Technology Centre. Because of limited space, the experiment is not described in this paper. The reader is referred to [3,47,48]. Generally, an active sensing system (LASER sensing system) was used, and two different sensor arrays were implemented called the INNER and OUTER sensor arrays; they consisted of six and seven sensors, respectively, and an actuator was used in each of them. The main motivation of the experiment was to implement a variety of different sensor techniques and find capable features that, using advanced machine learning tools, could detect damage at early stages before visual abnormalities were presented.

The approaches to be discussed fall into the category of pattern recognition that deals with the problem of deciding whether the features taken either from raw or analysed signals have arisen from a damaged or undamaged structure. *Supervised learning approaches* to pattern recognition include Bayesian classification methods, nearest-neighbour search, artificial neural network classifiers [49] and more recently support vector machines [50]. *Unsupervised learning approaches* have perhaps received less attention [1]; an example can be found in reference [51] where one-class support vector machines were used. In practice, however, it is not easy to apply supervised learning techniques owing to the lack of training data required for the models corresponding to damaged systems, which is why unsupervised learning/novelty detection approaches might probably be of more use for SHM or CM.

One of the aforementioned studies related to the experiment shown in figure 3 is given in reference [3], where a group of machine learning techniques for the monitoring of turbine blades by using vibration data and specifically high-frequency response function measurements (FRFs) was presented. The algorithms were optimized to an extent that could offer a fast and satisfactory online monitoring. In order to obtain features which are sensitive to damage, a dimensionality reduction of the FRF data was performed using probabilistic principal component analysis. Then, for the purposes of novelty detection (unsupervised learning), a trained five-layer auto-associative neural network was used to model the normal condition. An additional approach adopted in that paper was the use of a radial basis network (RBF) that was implemented in terms of auto-association. In practice, the advantage of the second approach adopted was that RBF networks when compared with multi-layer perceptrons do not usually need a full and challenging nonlinear optimization of all the parameters. Finally, a distance measure for novelty detection was calculated by feeding the two different network algorithms with testing data.

Apart from the vibration-based analysis in this case, other kinds of data that were obtained by this experiment were also analysed for damage detection purposes, although not necessarily appropriate for online strategies. In [52], there is a further investigation of post-processing of the data, by using digital image correlation snapshots taken along the length of the blade via a stitching technique. This allows observations of the shape and curvature of the entire blade and demonstrates that the technique can be scaled for utility-scale wind turbine blades.

## 3. An exploration of supervisor control and data acquisition system data of an offshore windfarm for condition and health monitoring

The use of SCADA data for monitoring has been shown in several studies, such as in [53–56], and in most cases it aims at the development of a complete and automatic strategy for the monitoring of the whole turbine or wind farm, although subcomponents (e.g. bearings, generator) may also be individually assessed. Among the various approaches, power curve monitoring has been popular and successful. Wind turbines have been designed by manufacturers to have a direct relationship between wind speed and the power produced, and as they require a minimum speed to produce the nominal power, but limit the power generated from higher wind speeds, the power curve usually resembles a sigmoidal function. A critical analysis of the methods for modelling the power curve can be found in [57], but, in general, researchers have exploited the deviation from a reference curve to perform SHM on turbines. The use of machine learning approaches for the estimation of power generation can be seen as far back as in [58,59], with more recent works appearing as well [60,61]. In [62], a steady-state model of a whole wind farm with neural networks was shown to have fair results if the data used were pre-processed, whereas in reference [63], three operational curves, power, rotor and pitch, were used for reference in order to produce control charts for the monitoring of wind turbines.

**Figure 4.**
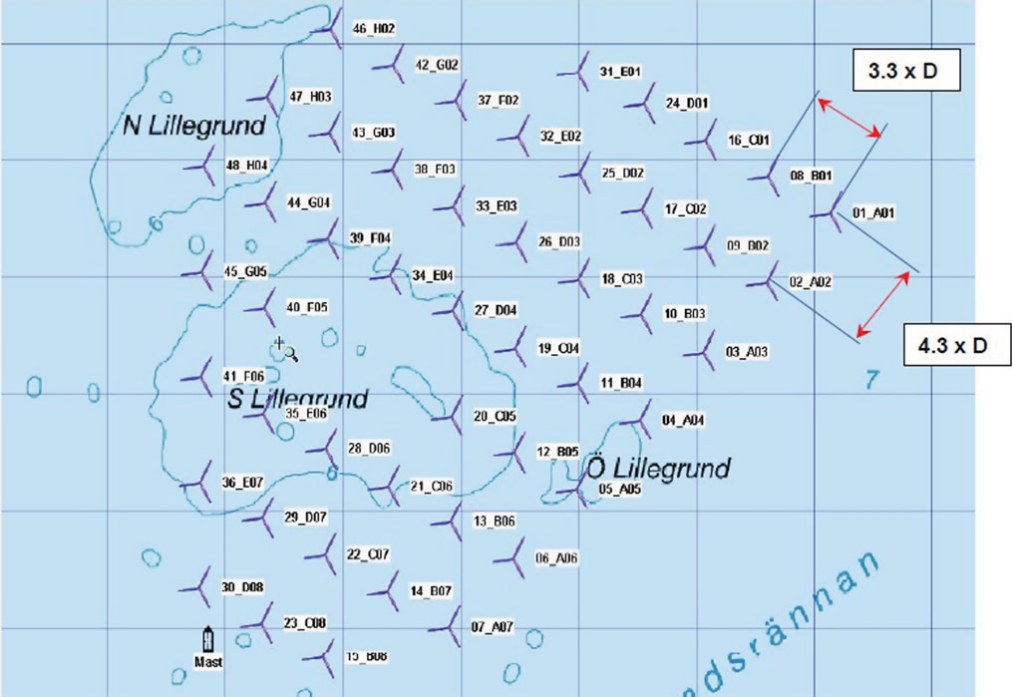
Lillgrund wind farm and the distribution of the wind turbines [65].

In the following, a primary exploration of SCADA data, found in reference [64], is going to be presented. It can be seen as a first step towards implementing a population-based approach for SHM in wind farms. The concept behind what we refer to as a population-based approach is that assuming a homogeneous population, we have the possibility of modelling one structure and using it for diagnosis on all similar structures. Any damage state data (which are always rare) becomes relevant to the whole population. The datasets used describe the fully operational Lillgrund wind farm that is situated in the sea area between Denmark and Sweden, consisting of 48 wind turbines of rated power of 2.3 MW [65] and is shown in figure 4. The wind turbines are Siemens SWT-2.3-93, characterized by a rotor diameter of 92.6 m and a hub height of 65 m, giving a rated power of 2.3 MW. The maximum rated power is reached when wind speeds take values of 12 m s^−1^ (rated wind speed). The spacing between the turbines in the specific wind farm is significantly closer than most conventional farms, and this unique element is generally expected to affect their performance. This wind farm architecture was done deliberately for analysing the effects and the interactions of each wind turbine within such close spacing.

Data mining and machine learning are promising approaches for modelling wind energy aspects such as power prediction or wind load forecasting. The complete study presented in this section can be found in [64]. Artificial neural networks and Gaussian processes were used to build a reference power curve (wind speed versus power produced) for each of the 48 turbines existing in the farm. Then, each reference model was used to predict the power produced in the rest of the turbines available, thus creating a confusion matrix of the regression model errors (MSEs) for all combinations. For the neural networks, multi-layer perceptrons were used. In addition, the reference power curve is a healthy power curve, i.e. built only using data corresponding to time instances with a status code equal to ‘0’ (‘no error’ in the turbines). The results showed that nearly all models were very robust with sensibly low MSE errors.

The available data used in this study correspond to a full year of operation. All the SCADA extracts consist of 10 min averages, with the maximum, mean, minimum and standard deviation of the 10 min intervals being recorded and available. The actual sampling frequency is less than 10 min.

**Figure 5.**
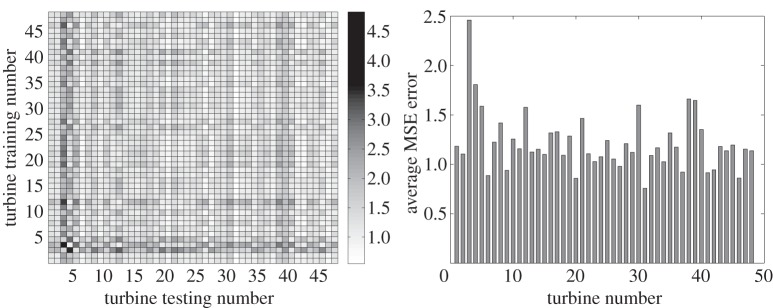
Results of the population-based approach using neural networks on the wind farm SCADA data. (*a*) Confusion matrix with MSE errors created from the neural networks—testing set. (*b*) Average MSE error showing how well neural networks trained to predict the power produced in each turbine, predict the produced power in the rest of the turbines.

**Figure 6.**
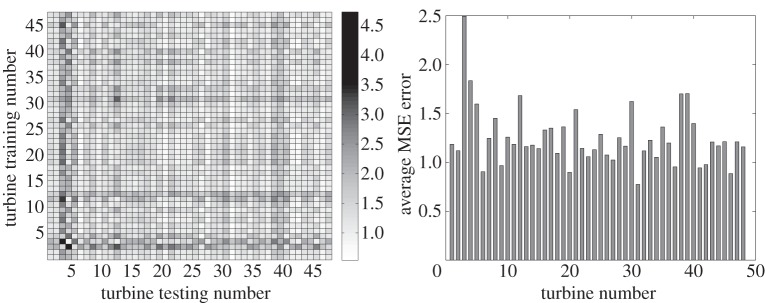
Results of the population-based approach using Gaussian processes on the wind farm SCADA data. (*a*) Confusion matrix with MSE errors created from the Gaussian processes—testing set. (*b*) Average MSE error showing how well each turbine (power produced) is predicted by the others—Gaussian processes.

In figures 5*a* and 6*a*, the confusion matrix created from the neural networks and the Gaussian processes testing sets is shown. Each axis of the confusion matrix shown corresponds to one up to 48 turbines, where on the *y*-axis is the number of the trained turbine and on the *x*-axis the number of the tested turbine. In general, an MSE error below 5 is considered a good fit and below 1 excellent. The results appear to be very good with the worst results being for turbines 3 and 4. These are the turbines that have the highest MSE error. According to Papatheou *et al*. [64], these errors are high owing to stops (emergency or manual) of these specific turbines for the majority of instances when the high error is observed. In terms of the comparison between neural networks and Gaussian processes, it appears that the results presented in the paper are very similar to the networks performing with a slightly lower MSE error. It should be noted that the Gaussian processes were trained with about one-third of the data that the neural networks were provided, but the testing sets are everywhere the same.

Figures 5*b* and 6*b* simply show the average MSE errors contained in the confusion matrices shown in figures 5*a* and 6*a*. The generally very low MSE errors show that the power curves have the potential of being used as a feature for the monitoring of the whole farm, as they were shown to be generally robust to the individual differences that the turbines inevitably present (location, different sensors, different generators, etc.). So, it can be seen how well the power produced in each turbine is predicted by the rest of the trained curves (corresponding to the rest of the turbines). Such analysis is a necessary first step in establishing novelty detection between individual machines.

## 4. Conclusion

This paper discusses some of the latest advances in the SHM and CM of wind turbines. In order to achieve an effective damage detection strategy, the use of different kinds of data is needed, depending on the approach. Data-driven vibration-based analysis methods seem to be able to provide such solutions, although difficulties exist related to the operational conditions of wind turbine systems. In this case, the resulting non-stationarity should be taken into account and it is only more sophisticated signal processing approaches such as time-frequency analysis, or co-integration that could successfully perform the feature extraction part of a complete SHM or CM procedure. For certain wind turbine components, such as the blades, the choice of an applicable and reliable sensing system is of great importance, and for this reason, some of the existing technologies were presented. Finally, pattern recognition and machine learning approaches can not only be useful for the feature discrimination part of the SHM procedure, but also for the manipulation of SCADA data. The potential behind the last concept was demonstrated in the previous section of this paper, where it was shown on actual wind farm data that a population-based approach towards wind turbine SHM might be a successful choice.
